# Paying It Forward: Generalized Reciprocity in Mass Opinion on Foreign Aid

**DOI:** 10.1093/poq/nfag003

**Published:** 2026-03-19

**Authors:** Joonbum Bae, Changkeun Lee

**Affiliations:** Assistant Professor, Department of Political Science, Colorado State University, Fort Collins, CO, US; Associate Professor, KDI School of Public Policy and Management, Sejong, South Korea

## Abstract

Does providing American vaccines overseas improve views of the United States? Do beneficiaries of donated shots change their opinions on foreign aid? Can the example of the United States providing aid lead to higher support for international assistance in other nations? Utilizing original panel data from a two-wave survey fielded in South Korea in 2021 and 2022, this paper finds no evidence that American shots, whether donated or purchased, lead to more positive views of the United States. However, we document a “pay it forward effect,” where recipients of donated COVID-19 vaccines from the United States were more likely to pass on the generosity by supporting *Korean* vaccine aid to other countries in need. Information that the United States was supplying assistance to developing countries also made it more likely that vaccinated South Koreans would support their government donating vaccines abroad. This study provides evidence of second-order effects of vaccine aid that can benefit American interests by facilitating the timely distribution of vaccines across the globe, even when it does not improve the donor’s image. The results highlight the role foreign aid can play in furthering international cooperation and call for broader criteria when evaluating its effect.

## Introduction

Samantha Power, former administrator of the US Agency for International Development, argued that “vaccines in arms” were the way to restore American leadership damaged during the Trump years. “U.S. provision of lifesaving aid,” she claimed, would “have a significant positive effect on American standing internationally.” With vaccines scarce globally but plentiful in the United States, sending shots abroad could serve as a powerful tool of American statecraft (Power 2021).

Foreign vaccine aid can serve American interests in several ways. First, as Power contends, American ingenuity in developing vaccines or the generosity behind their free provision can improve the US image and bolster its status among recipient nations, enhancing Washington’s global standing. Yet the link between foreign aid and American standing abroad remains unclear (Goldsmith, Horiuchi, and Wood 2014; Dietrich, Mahmud, and Winters 2018; Mattingly and Sundquist 2022).

Second, as this paper outlines, contributing vaccines abroad can further US interests by facilitating international cooperation on the global supply of vaccines. Protection against the virus from gifted shots can increase public support in receiving countries for vaccine aid to *other* nations. This boost in support for “paying it forward” can spur subsequent shipments of jabs from these nations to a third set of countries, helping meet global vaccine demand. Delivering immunity to another country can positively influence the pandemic’s trajectory via such a “vaccine cascade.” The outcome from an initial round of aid, therefore, could be international cooperation based on generalized or indirect reciprocity, where actors receiving help do not return the favor but pass on generosity to support others in need (Putnam 2000; Diekmann 2004; Nowak and Sigmund 2005; Bartlett and DeSteno 2006; Stanca 2009; Van Doorn and Taborsky 2012; Gray et al. 2014).

Finally, awareness of the United States providing aid to other countries in need can further galvanize support for foreign (vaccine) aid among the public in third-party nations. Scholarship has stressed the influence of normative considerations on foreign affairs. Ideas, values, and principles from other countries can raise the standards of appropriateness and win adherents abroad, ultimately leading to changes in policy and political outcomes. Knowledge of American assistance could exert a normative pull on the peoples of other nations, with the public abroad in third-party countries becoming more supportive of aid through an “example effect” (Meyer and Rowan 1977; Finnemore and Sikkink 1998; Dobbin, Simmons, and Garrett 2007; Nye 2008).

These three pathways are not mutually exclusive. It is an empirical question which, if any, are relevant for foreign aid in general and Washington’s response to the COVID-19 pandemic. Does the provision of American vaccines abroad improve US standing? Could being vaccinated with donated shots influence opinions regarding foreign aid? Can the example of US aid lead to higher support for international assistance in other nations?

This paper addresses these questions using original panel data from a two-wave survey in South Korea in 2021–2022. South Korea purchased American shots as part of its COVID-19 response and also received US vaccine aid, enabling direct comparison of their effects. The thirty-year age cutoff the Korean government applied to the donated shots ahead of the 2021 survey facilitates inferences about the effect of American aid on both opinions of the United States and support for South Korean vaccine aid. Survey experiments in the second wave further probe the mechanisms behind a potential “example effect” of vaccine provision on mass opinion.

We find that the free provision of vaccines from Washington had no discernible effect on Korean recipients’ views of the United States. Shots procured independently by the South Korean government from American vaccine companies also did not influence views of the United States. Neither American competence in developing vaccines nor the generosity of their provision affected opinions of the United States.

There was, however, evidence that respondents who had received donated American jabs were more likely to support *South Korean* vaccine aid to other developing countries. Even though the same shots did not improve Washington’s standing among the Korean public, beneficiaries from American assistance were willing to “pay it forward” by backing vaccine aid for other countries in need.

Moreover, support for foreign aid increased when survey respondents were presented with information that the United States was providing vaccine aid to developing countries, even when they had not benefited directly from US assistance. The example of the United States providing vaccine aid to third parties in need bolstered support for South Korea giving aid abroad. However, this “example effect” was limited to political moderates, who numbered about half the respondents in the survey.

This paper contributes to the scholarly literature by theorizing and testing distinct pathways through which foreign aid can impact the national interests of donor countries. Findings on a “pay it forward” effect as well as an “example” effect to foreign aid add to our understanding of the factors behind support for international assistance (Zürcher 2012; Goldsmith, Horiuchi, and Wood 2014; Tokdemir 2017; Dietrich, Mahmud, and Winters 2018; Green-Riley 2020; Dolan and Nguyen 2021; Kobayashi, Heinrich, and Bryant 2021; Kohno et al. 2021; Schneider et al. 2021; Sharun and Dhama 2021; Mattingly and Sundquist 2022) and vaccination’s impact on opinion (Lee 2023).

The results documented in this paper also advance our understanding of how international cooperation can emerge from the context of external shocks such as a pandemic. Prior research examining the impact of disasters and emergencies has focused chiefly on their adverse effects on relations between countries and domestic stability (Grasse et al. 2021; Lee, Mitchell, and Schmidt 2022; Barceló et al. 2022; Reinhardt and Lutmar 2022; Wood et al. 2022; Brancati, Birnir, and Idlbi 2023). Others argue that emergency aid can lead to an improvement in relations with the donating nation (Ganapati, Kelman, and Koukis 2010; Akcinaroglu, DiCicco, and Radziszewski 2011). To our knowledge, no study has examined whether recipients of foreign aid are more willing to support *third parties* in need. By presenting evidence of such generalized reciprocity in foreign aid, this paper points to a novel source of international cooperation: the people’s willingness to pay generous behavior forward.

In terms of policy, the potential for a chain of goodwill, where destination states of vaccine aid donate vaccines to other nations via a “pay it forward” effect, challenges decision-makers to devise innovative strategies for vaccine allocation during a fast-spreading epidemic in ways that can serve the national interest as well as the global public good. The example set by US donations encouraging individuals abroad to support their own country’s aid also reshapes our understanding of the most effective communication strategy to accompany foreign aid. More generally, the results call for broadening the criteria for evaluating foreign aid by highlighting ways American aid can further its interests without necessarily boosting affinity toward the United States.

## Generalized Reciprocity, International Cooperation, and Foreign Aid

### Generalized Reciprocity and Cooperation

How rational actors can cooperate in the absence of a higher coercive authority has been a central issue in the study of international politics. Scholars have often turned to reciprocity, deeming it the “most effective strategy for maintaining cooperation among egoists” (Keohane 1986). Axelrod famously argued that cooperation was possible via a strategy of “tit for tat” when states interacted under the long shadow of the future (Axelrod and Hamilton 1981).

However, the question remained whether cooperation could emerge when direct reciprocation was unlikely. In international politics, for example, where resource asymmetries between states are often significant and enduring, reciprocal cooperation is often impractical or impossible; therefore, it is unclear whether states have a reason to collaborate.

Outside of relations between states, in contrast, many have long argued that cooperation was possible even without direct reciprocation, regardless of an imbalance of resources. Collaborative relations within a group could form based on the simple but powerful logic of “giving something if you have received anything.” Actors did not need to have commensurate levels of resources to cooperate; one just needed to extend an act of generosity that others would pass on in some way. Such “indirect” or “generalized” reciprocity has been applied to explain cooperative behavior in various studies in the social and natural sciences but has yet to be applied to international aid (Diekmann 2004; Bartlett and DeSteno 2006; Rutte and Taborsky 2007; Stanca 2009; van Doorn and Taborsky 2012; Chiang 2021).

Different motivations have been posited for why actors engage in cooperation through generalized reciprocity. On the one hand, the rationalist view posits that actors do so for long-run benefits. The logic was that “I’ll do this for you without expecting anything specific back from you, in the confident expectation that someone else will do something for me down the road” (Putnam 2000). If the sequence of benevolence eventually comes full circle, all members could benefit from a chain of collaboration within the group.

Alternatively, norms could provide the foundation for beneficiaries of generosity to pass on goodwill. Actors do so not because they expect material benefits but based on a social understanding that it is appropriate or right. A widely shared sense of the obligatory nature or appropriateness of “paying it forward” could nevertheless result in cooperative acts becoming established among large numbers of actors even without expectations of future returns.

### Foreign Aid and Generalized Reciprocity

Despite the social and rational motivations for paying it forward, there are incentives for actors not to repay the generosity that they receive from others, particularly when the stakes involved are high. Thus, the rationalist account of generalized reciprocity has often required specifying the scope conditions (the number and nature of actors or an enforcement mechanism) under which it would be optimal to engage in generalized reciprocity (Keohane 1986; Nowak and Sigmund 1998; Pfeiffer et al. 2005; Rankin and Taborsky 2009).

Similarly, even if deeply held norms favor “paying it forward,” if the costs of doing so (or the benefits of not doing so) are large, the “logic of consequences” may override the logic of appropriateness (March and Olsen 1989). Norms that have reached a taken-for-granted status can also run against their limits as actors face higher costs to uphold (Press, Sagan, and Valentino 2013; Kertzer and Rathbun 2015). States are unlikely to adhere to normative codes when they entail severe consequences (Diekmann 2004).

Foreign aid in response to acute emergencies, however, offers the promise of collaborative generalized reciprocity even on issues of life and death. This is partly due to the transitory nature of crises. A crisis creates dire needs for emergency aid, and the benefits from receiving and distributing the aid are substantial. It is challenging for states under such duress to assist others, even when strong norms supporting generalized reciprocity are deeply ingrained in society.

The incentives to not pass on goodwill, however, diminish as the crisis conditions behind the hardship recede. Countries receiving emergency relief after natural disasters may be more than willing to provide aid to other nations in need after rebuilding. Patients who have received a blood transfusion cannot immediately donate blood. Nevertheless, many volunteer to do so after recovery (Ferguson 2021). Similarly, countries experiencing vaccine shortages during a pandemic are unlikely to have the means to send vaccine aid to other nations, but could support doing so once herd immunity is reached.

Thus, lifesaving foreign aid during transient crises can encourage future rounds of aid from the initial beneficiaries and thereby enhance cooperation between states via indirect reciprocity. The dissipating need for aid makes it easier for initial receivers of assistance to pass on the generosity to others in need. The extension of help during acute periods of need is also more likely to mobilize norms of positive generalized reciprocity and bolster public support for “paying it forward” once the initial crisis conditions have passed. In theory, this process can perpetuate itself, and the number of actors involved in paying generosity forward can increase at each stage of giving, leading to cascades of assistance across the state system.

Although not essential for cooperation via generalized reciprocity, aid can also have positive externalities that benefit the sending countries. Vaccine aid during a pandemic benefits not only those receiving the shots but also others, including those in the nation providing aid, as vaccinations reduce disease transmission and can help bring the pandemic under control. Providing protection to others at a sufficient scale also reduces the likelihood of a virus mutation and the further spread of infections.^1^

## South Korea and American Vaccine Aid

South Korea meets several key conditions for assessing the effect of vaccine aid on mass opinion. While Washington primarily targeted developing countries with its vaccine aid, it made an exception for Seoul just ahead of the May 2021 US–Korea summit meeting, delivering just over 1.4 million Johnson & Johnson (J&J) doses in June 2021.

At the time, fewer than two million people—mainly the elderly, health workers, and high-risk groups—had received any COVID-19 vaccine in Korea.^2^ Younger age groups and healthy adults faced months of waiting under the national vaccination program’s priorities. To those eligible, the single-dose J&J vaccines offered immediate protection when no alternatives were available. The highly publicized donation announced just before the summit meeting during a period of acute vaccine shortages attracted substantial media coverage and public interest. This ensured that many recipients were aware that the vaccines came from the United States, creating an unusual opportunity to study the attitudinal effects of US aid.^3^

At the same time, Seoul’s 2021 vaccination program simultaneously utilized shots purchased on the international market and those received through bilateral aid, allowing for the direct comparison of the effects from these two sources. Unlike in many countries, political elites in Korea shared a broad consensus on the efficacy and necessity of vaccines, avoiding partisan divides that could confound opinion shifts (Besch, Korn, and Bohm 2021; Pink et al. 2021; Hwang, Kim, and Heo 2022). South Korea is also a relative newcomer to international aid donor status, having received foreign aid as late as the 1990s, and will be of interest to those who study the factors behind public support for foreign aid in industrialized democracies.

Finally, by agreement between Seoul and Washington, the donated shots were reserved for South Koreans in national-defense-related fields. Due to South Korea’s conscription policy, all men complete mandatory military service in their early twenties and go on to take on roles in the reserves or civil defense in their thirties to forties.^4^ The government excluded those under thirty from receiving J&J due to concerns about rare side effects, mainly leaving men in their thirties and forties eligible for the donated shots. The eligibility cutoff that the government policy created at age thirty makes it possible to make stronger claims about the causal effect of aid.

## Survey Data

Embrain, an online polling firm, conducted the panel surveys used in this study.^5^ The surveys sought to secure variation in vaccination status across respondents to estimate the causal effects of vaccination via before-and-after differences. At the sampling stage, we attempted to approximate the general population through stratification based on age, gender, and residential location. The first wave yielded a total of 3,459 responses, with 2,006 participants proceeding to the second wave. All subsequent analysis uses the unweighted sample.

The first survey was conducted in August 2021, when vaccines were still relatively scarce in South Korea. According to data released by the Korean government, about 40.8 percent of the population received at least one dose as of August 6. Table 1 shows that our survey data is representative of the population in terms of vaccination rates.

**Table 1.** Vaccination rates, official announcement and our data.

	% 1st dose in Aug 2021			% Full vaccination in Jan 2022	
	Official stat (Aug 6 2021)	Our data	Our data-J&J	Official stat (Jan 6 2021)	Our data
20s	26.0	28.8	0.0	94.6	94.4
30s	30.3	39.4	15.7	91.2	93.0
40s	25.6	33.8	4.0	92.8	94.2
50s	39.4	43.1	1.3	96.2	95.1
60s and older	87.3	84.7	1.9	94.7	92.7

Due to the government’s prioritization of the older population in the early stages of the vaccine program, there were considerable differences in the vaccination rate across age groups in the first survey. The rate ranged from 87.3 percent for those aged sixty and older to approximately 30 percent for those aged forty years and younger. The government’s vaccination effort also prioritized medical and healthcare workers, teachers, workers in critical industries such as semiconductors, motor vehicles, and steel, as well as those with preexisting conditions. As a result, most vaccinated people under fifty in the first survey fell into one of these categories.

The second-wave survey was conducted in February 2022, when over 85 percent of Koreans twelve and over were *fully* vaccinated.^6^ By then, almost everyone who wanted a jab had received one. Since those who refused to be vaccinated could have traits correlated with views about the United States and vaccine aid policy, we utilize a difference-in-difference design by comparing the shifts in opinions between those vaccinated in both surveys and those vaccinated between the first- and second-wave surveys. We also employ cross-section analysis with the first-wave data, when most of the population was still unvaccinated, to cross-check results from the difference-in-difference design.

To test for the “pay it forward effect” of American vaccine aid, we leverage the discontinuity inherent in Seoul’s thirty-year age cutoff in determining eligibility for the J&J donated vaccines. We do so by comparing the views of those immediately to either side of the government’s eligibility criteria in the first survey.

Finally, we randomly assigned respondents in the second survey information regarding American aid to the developing world and observed the difference between the treatment and comparison groups. This setup was designed to test for a possible “example effect” that American aid might have on those who are aware of it. Since about 95 percent of adults were fully vaccinated at this point, the scope of this example effect was limited to those already protected from the virus.

## The Effect of Vaccination on Foreign Policy Opinion

### Vaccination and Views of the United States

We first examine whether vaccination improved opinions of the United States. While most vaccines in the Korean government’s vaccination program—Pfizer, Moderna, and Johnson & Johnson (J&J)—were developed by American companies, the United Kingdom’s AstraZeneca (AZ) jab accounted for a substantial proportion of early vaccinations. At the time of the first survey in August 2021, AZ accounted for about 37.8 percent of all shots. However, as American vaccines later became the norm in the vaccine rollout, AZ’s share dropped to 21.8 percent by the second-wave survey.

Because our goal is to estimate the causal effect of vaccination, an ideal setting would have as the treatment group those randomly vaccinated between the two waves. The control group would remain unvaccinated in the second wave, independent of their willingness to get a shot. However, because individuals select into vaccination status contingent upon priorities assigned by the government, the more vaccine rates go up, the more the control group is populated by people unwilling or unable to receive vaccines. Therefore, inferences based on comparing those who remained unvaccinated with those vaccinated are problematic.

Due to the accelerated pace of vaccination, about 95 percent of eligible Korean adults were *fully* vaccinated when the second survey was conducted in February 2022, leaving a relatively small unvaccinated control group.^7^ Within this group, a substantial proportion were likely to be opposed to vaccination or unable to receive the shot due to health reasons. Since refusal of the jabs or health conditions that prevent inoculation could be correlated with opinions on foreign vaccine aid and the country donating the vaccines, a research design that compared the views of the newly vaccinated with those who remained unvaccinated could result in biased conclusions.

We take a different approach to this identification problem by setting the control group as respondents fully vaccinated by the first wave and the treatment group as those who received the vaccine between the first and second surveys. Doing so limits the scope of analysis to those fully vaccinated by the second wave. As table 1 shows, 95 percent of respondents were fully vaccinated by the second survey. The approximately 5 percent of those surveyed who chose not to be vaccinated are dropped from the analysis. This somewhat limits our ability to make population-level inferences from the data. However, it also substantially reduces the chances of bias from relying on respondents who opt out of vaccination to reach conclusions about its effect.

We consider the following first-difference regression specification for regression analysis:


$$\Delta y_{it}=\beta_{0}+\beta_{1}\Delta {\mathrm{Vaccine}}_{\mathrm{it}}+\Delta \epsilon_{it}$$

where $y_{i}$ denotes the dependent variable, a Likert-scale indicator of how much respondent *i* is favorable toward the United States (1: very unfavorable to United States, 7: very favorable to United States). $\beta_{1}$ is the coefficient of our interest. $\Delta {\mathrm{Vaccine}}_{i}$ is the change in vaccination status. It is equal to 1 if the individual was not vaccinated in the first survey and fully vaccinated in the second survey.

In the regressions, we include several control variables: gender, age group, educational attainment, and political orientation. Political orientation is a categorical variable derived from the original seven-point self-placement scale. Respondents selecting 1–3 are coded as “conservative,” 5–7 as “progressive,” and 4—the most frequent response in our sample—as “moderate” (the reference category). This coding reflects the strong centrist tendency in Korean political self-identification, with over 50 percent of respondents selecting 4. Age and education are also measured as categorical variables: age is grouped by decade (twenties, thirties, etc.), and education ranges from less than high school (coded as 1) to postgraduate education (coded as 5). Descriptive statistics for these variables are presented in table 2.

**Table 2.** Descriptive statistics (for those who responded in both waves).

Variable	# Observations	Mean	SD
Vaccinated in the first wave (both partial and full)	2,006	0.444	0.497
Fully vaccinated in the second wave	2,006	0.940	0.237
Vaccinated after 1st wave	2,006	0.504	0.500
Female	2,006	0.439	0.496
Age	2,006	44.7	12.3
Political orientation (1–7)	2,006	4.126	1.097
View of the US (1–7)			
1^st^ wave	2,006	4.284	1.195
2^nd^ wave	2,006	4.262	1.257
Change	2,006	−0.022	1.002
Support for vaccine aid (1–7)			
1^st^ wave	2,006	4.262	1.257
2^nd^ wave	2,006	4.353	1.620
Change	482	0.670	1.528

Table B2 reports results from eight tests of the hypothesis that American vaccines improve favorability toward the United States. We found no evidence supporting this hypothesis across specifications that utilize cross-sectional analysis (of both waves), over-time changes in opinions leveraging the panel data, and the subsetting of age groups according to government vaccine policy. For the recipients of these potentially lifesaving shots, the innovative capacity and competence behind their development did not boost the standing of the country where they originated.^8^

Could these results be due to ignorance about the national origin of the vaccines? This is a concern, particularly considering research on foreign aid that has shown that large majorities in recipient nations are unaware of the origin of aid, even when directly presented with information about the donor country (Dietrich, Mahmud, and Winters 2018).

In the first survey, we asked vaccinated respondents an open-ended question regarding the national origin of the vaccines they had received. For all the American vaccines (Moderna, Pfizer, and J&J), the correct identification rate was about 80 percent. Less than 20 percent either misidentified the country or wrote that they did not know. The most frequent case of misidentification involved responses that incorrectly identified American vaccines as British. Such cases accounted for approximately 6 percent of responses. Table B3 presents this information. The substantial majorities that specified in an open-ended question that Pfizer, J&J, and Moderna were American vaccines, as well as the low number of respondents that misidentified the national origin of these products, gives added confidence that the null result is unlikely to reflect ignorance of vaccine origin.

As a robustness check, we repeated the analysis using a restricted sample of vaccinated respondents who correctly identified the national origin of their vaccine. In this regression, we compare those vaccinated with correct knowledge of their vaccine’s origin to those not vaccinated. The results in table B4 broadly support our main findings: receiving a US-produced vaccine did not lead to more favorable views of the United States, even among respondents aware of its origin.

While attrition between waves was not random—women, younger, and less educated respondents were more likely to drop out—a *t*-test showed no significant difference in baseline views of the United States between those retained and those lost to follow-up (*p *= 0.46). This suggests that our main outcome measure is unlikely to be biased by panel attrition.

### Immunity and Support of Vaccine Aid

Although the provision of American vaccines does not positively influence perceptions of the United States, it could have other effects relevant to American foreign policy goals. This section tests the hypothesis that vaccine recipients become more supportive of their government sending vaccines abroad. Koreans immunized with American shots, in other words, could turn more positive toward sending *Korean* vaccine aid abroad. When still waiting for shots, respondents are unlikely to support vaccines that could be used domestically going to another country. After gaining immunity, however, the vaccinated are more likely to turn their attention to those who cannot protect themselves from the virus. They may be even more supportive of sending aid when they have benefited from the generosity of donors abroad.

To measure respondents’ views on Seoul giving vaccine aid, our survey asked to what extent they “agree that Korea should provide vaccine aid to developing countries for humanitarian purposes.” The responses were recorded on a seven-point Likert scale (1: Strongly disagree, 7: Strongly agree). The question did not designate receiving countries or the number of potential destinations for the aid, except to specify that aid would be for developing nations. The purpose of the aid was also characterized as humanitarian. Such wording was intended to minimize respondents’ expectations that Korea would receive something in return for the aid and, therefore, to exclude direct reciprocity as a motivation for the support for aid. We adopt the same regression specification as the previous subsection.

In the second survey, we test for the “example effect” in support for international assistance. Scholars in various fields have theorized about how the actions and expectations of others can influence the behavior of actors (Bandura and Walters 1977; Mead 2015). In the international realm, awareness of the United States donating aid to the developing world could exert a normative pull on the citizens of other nations, making them more likely to support aid from their own country (Nye 2008; Finnemore and Sikkink 1998; Hattori 2001; Ingebritsen 2002). To test for this effect, we introduce an experiment in the second-wave survey. For some respondents, we randomly embedded information about US aid policy just before the question on support for Korea providing vaccine aid to the developing world. The embedded passage states: “The US is providing aid to the developing world for humanitarian purposes.” For the control group, nothing was added to the question. Then, we observe the difference between the treatment and comparison groups’ support for Korean assistance to other nations. Since 95 percent of adults were fully vaccinated by the second survey, any effect of the information treatment would be driven by those with immunity. This experimental design was not preregistered. Hence, the results should be considered exploratory in nature.

We first report the cross-sectional results in table 3. Vaccination status is associated with more positive views of vaccine aid to developing countries in the first wave. This effect disappears in the second wave (in columns 5 and 6). As noted above, about 95 percent of the respondents were vaccinated by the second survey, leaving a small percentage of the respondents who were likely to be unable or unwilling to receive the vaccine as the comparison group.

**Table 3.** Vaccination and opinion on vaccine aid: cross-section.

	(1)	(2)	(3)	(4)	(5)	(6)
Dependent variable:	Support for vaccine aid (1–7)					
Data:	1^st^ wave			2^nd^ wave		
Sample:	All	All	Under 60	Under 60	All	All
Vaccinated	0.299	0.204	0.309	0.180	0.037	0.178
	(0.000)	(0.114)	(0.000)	(0.223)	(0.825)	(0.340)
Vaccinated		0.132		0.168	0.103	0.103
x US vaccine		(0.339)		(0.285)	(0.191)	(0.193)
Information cue						−0.220
about US vaccine aid						(0.110)
Female	−0.318	−0.316	−0.349	−0.345	−0.184	−0.182
	(0.000)	(0.000)	(0.000)	(0.000)	(0.023)	(0.024)
Age						
20s	0.066	0.073	0.073	0.082	0.272	0.276
	(0.594)	(0.558)	(0.556)	(0.508)	(0.071)	(0.067)
30s	0.594	0.605	0.598	0.612	0.659	0.663
	(0.000)	(0.000)	(0.000)	(0.000)	(0.000)	(0.000)
40s	0.638	0.644	0.643	0.652	0.902	0.900
	(0.000)	(0.000)	(0.000)	(0.000)	(0.000)	(0.000)
50s	0.749	0.826			0.871	0.756
	(0.000)	(0.000)			(0.000)	(0.000)
Education						
Some college	0.073	0.074	0.065	0.066	0.080	0.077
	(0.411)	(0.405)	(0.507)	(0.498)	(0.453)	(0.466)
Postgraduate	0.234	0.234	0.219	0.221	0.204	0.202
	(0.114)	(0.114)	(0.189)	(0.185)	(0.154)	(0.159)
Political orientation						
Conservative	−0.655	−0.656	−0.761	−0.764	−0.151	−0.151
	(0.000)	(0.000)	(0.000)	(0.000)	(0.180)	(0.183)
Progressive	0.361	0.362	0.370	0.371	0.442	0.443
	(0.000)	(0.000)	(0.000)	(0.000)	(0.000)	(0.000)
Constant	3.609	3.599	3.638	3.627	3.986	4.057
	(0.000)	(0.000)	(0.000)	(0.000)	(0.000)	(0.000)
Observations	1,733	1,733	1,500	1,500	1,006	1,006
*R*-squared	0.102	0.102	0.105	0.106	0.111	0.113
*F*-test value	18.679	17.061	18.505	16.974	11.598	10.859

*Note*: Unstandardized regression coefficients are reported. Parentheses contain *p*-values from robust standard errors.

We also find from the second survey that the informational cue of the United States giving vaccine aid to developing countries does not make the respondents more supportive of South Korean aid (columns 5 and 6). The coefficient for the dummy variable indicates that the information treatment is not statistically significant.

With first-difference estimation, examining the effect of *new* vaccinations on *changes* in views of vaccine aid, we again find that receiving American shots generally does not increase support for vaccine aid originating from Seoul. Table B5 reports these results. They also show that presenting information on US aid to the developing world does not affect support for the Korean government sending vaccine aid abroad. This rejects the presence of an “example effect,” where knowledge that the United States is donating shots to the developing world boosts support for vaccine aid by the Korean government. It also suggests that simply being vaccinated is insufficient to increase overall support for sending vaccine aid abroad.

### Heterogeneity by Political Orientation

While there is little evidence that vaccination leads to a better image of the United States or higher support for vaccine aid, the results represent the average effect and may mask differential effects across the respondents. To examine potential heterogeneity, we repeat the same first-difference analysis with interaction terms between vaccination and political orientation. The results are in panel A of table 4. We also test for possible differential effects of information on US aid (from the second survey) contingent on political orientation and report the results in panel B. Only relevant coefficients are presented, and most control variables are omitted from the table for brevity.

**Table 4.** Heterogeneity by political orientation.

A. Vaccine effect				
	(1)	(2)	(3)	(4)
		
Dependent variable:	△ View of US		△ Support for vaccine aid	
Vaccinated after 1^st^ wave	−0.028	−0.030	0.236	0.254
	(0.670)	(0.670)	(0.203)	(0.177)
Conservative	0.136	0.126	0.672	0.722
	(0.108)	(0.142)	(0.009)	(0.007)
Progressive	0.054	0.058	0.175	0.165
	(0.495)	(0.465)	(0.491)	(0.524)
Vaccinated after 1^st^ wave x conservative	−0.023	−0.015	−0.335	−0.358
	(0.847)	(0.900)	(0.392)	(0.372)
Vaccinated after 1^st^ wave x progressive	−0.016	−0.012	0.029	0.043
	(0.885)	(0.909)	(0.931)	(0.900)

Demographic controls	No	Yes	No	Yes

Observations	1,886	1,886	452	452
*R*-squared	0.003	0.005	0.023	0.030
*F*-test value	1.090	0.880	1.772	0.993


B. Information cue effect (second wave)				
	(5)	(6)	(7)	(8)
		
Dependent variable:	View of US		Support for vaccine aid	
Conservative	0.764	0.764	0.065	0.065
	(0.000)	(0.000)	(0.694)	(0.695)
Progressive	0.110	0.109	0.674	0.676
	(0.389)	(0.391)	(0.000)	(0.000)
“US giving aid” statement	−0.189	−0.188	0.322	0.322
	(0.054)	(0.054)	(0.003)	(0.003)
“US giving aid” statement	0.162	0.162	−0.434	−0.433
x conservative	(0.441)	(0.442)	(0.051)	(0.052)
“US giving aid” statement	0.009	0.010	−0.465	−0.467
x progressive	(0.958)	(0.956)	(0.008)	(0.007)

Demographic controls	No	Yes	No	Yes

Observations	1,006	1,006	1,006	1,006
*R*-squared	0.129	0.118	0.119	0.121
*F*-test value	11.092	10.418	10.500	9.955

*Note*: Unstandardized regression coefficients are reported. Parentheses contain *p*-values from robust standard errors.

Panel A shows that even when accounting for diverging effects of political orientation, vaccines have no discernible effect on favorability toward the United States. In line with previous results, American shots in arms did not improve the standing of the United States. Nor did the provision of vaccines increase support for foreign aid. None of the estimated coefficients are significant.

A noteworthy result in panel A is that conservatives who were already vaccinated at the time of Wave 1—initially the least supportive group—exhibited the largest increase in support between the two waves. In the first wave, conducted during a period of vaccine scarcity, conservatives reported significantly lower support for vaccine aid (mean = 3.53) compared to moderates (4.02) and progressives (4.47). By the second wave, this gap had narrowed, primarily due to rising support among conservatives. Recall that individuals who were never vaccinated were excluded from the analysis, ensuring that these results compare attitudinal change between those who were always vaccinated and those who were vaccinated between the two surveys. This shift may reflect the broader improvement in the vaccine supply situation between the two surveys. As the perception of scarcity diminished, those with more reservations about sending vaccines abroad may have become more comfortable supporting international aid.

Panel B suggests that the information cue on US aid to the developing world has a positive effect on the views of centrists regarding South Korean vaccine aid. Centrists, as operationalized here, are those with responses of 4 on an ideological scale running from 1 (very conservative) to 7 (very progressive). Such political moderates number slightly over half of the respondents. Thus, there is evidence of an “example effect” to American leadership in vaccine aid, which spurs public support for further aid in third-party countries. This effect, however, is limited to political moderates or centrists.

Concomitantly, the example that the United States sets also negatively affects support for aid. Although progressives have a higher baseline level of support for foreign aid than conservatives or centrists, their backing for Korean vaccine aid drops significantly when exposed to information that the United States is providing vaccines to developing countries. The magnitude of the negative effect from progressives is such that it seems to offset any positive “example effect” of American vaccine aid to the developing world for the population as a whole. Attitudes toward the United States are a salient cleavage in the politics of many countries, and such “backlash” effects to public diplomacy based on the example of the United States may be relevant in contexts outside of Korea (Keohane and Katzenstein 2011; Goldsmith and Horiuchi 2007).

## American Vaccine Aid: Paying It Forward?

Providing shots abroad can strengthen support for vaccine aid among the inoculated when coupled with information about the United States donating shots to the developing world. However, countervailing effects contingent on political ideology limit the scope and magnitude of this effect. The analysis thus far did not attempt to isolate the effect of shots that the United States had donated from those that the Korean government procured on the market.

This section investigates whether *how* the United States provides vaccines impacts support for vaccine aid. Prior analysis did not reveal significant effects of being vaccinated on support for foreign aid unless information was provided on the United States sending aid to developing nations. Beneficiaries of American vaccine aid, however, could be more willing to “pay it forward” by supporting South Korean aid to others in need (even without information about US aid to other nations).

As described in Appendix C, the American government donated J&J vaccines to Korea when it faced a shortage of vaccines in early summer 2021. Because it is a one-shot vaccine and was administered to recipients before our first survey, most who received the J&J shot were already vaccinated by the time of the first-wave survey. This hinders us from employing the same first-difference specification.

Our alternative empirical strategy utilizes only the first wave and the discontinuity inherent in the government’s eligibility criteria for J&J vaccines. Only those thirty years and older were allowed to receive the J&J vaccine because of the blood clot risk—particularly among the young—associated with all vaccines using adenovirus vectors. This decision enables us to employ a discontinuity design, with eligibility cutoff at thirty. Limiting the sample to those between twenty-seven and thirty-two years old, we conduct tests with different specifications and report the results in table 5. In columns 1 and 4, we use eligibility for the J&J vaccine (where we code as equal to 1 if the individual is thirty years or older) to test whether belonging to the group for which the J&J vaccines were donated (without necessarily receiving the shot) raises their level of support for foreign aid. In columns 2 and 5, we test the effect of actual vaccination of both J&J and other vaccines. In columns 3 and 6, we instrument J&J vaccination by eligibility for the vaccine. Note that all the other vaccines (except AstraZeneca) were administered without a cutoff at thirty years of age. The “other vaccines” variable is simply included as a control variable in the models reported in table 5.

**Table 5.** Donated vaccines and views of the US.

A. All gender						
	(1)	(2)	(3)	(4)	(5)	(6)
		
Dependent variable:	View of US			Support for vaccine aid		
J&J eligibility	−0.026			0.548		
	(0.848)			(0.053)		
J&J vaccination		0.031			0.903	
		(0.864)			(0.018)	
J&J vaccination, instrumented			−0.104			2.583
			(0.847)			(0.060)
Other vaccines	−0.116	−0.112	−0.121	0.521	0.533	0.637
	(0.306)	(0.326)	(0.301)	(0.012)	(0.010)	(0.005)

Demographic controls	Yes	Yes	Yes	Yes	Yes	Yes

Observations	514	514	514	258	258	258
*R*-squared	0.138	0.138	0.137	0.133	0.132	0.087
*F*-test value	13.329	13.305		6.876	6.398	


B. Male only						
	(7)	(8)	(9)	(10)	(11)	(12)
		
Dependent variable:	View of US			Support for vaccine aid		
J&J eligibility	−0.059			0.656		
	(0.663)			(0.028)		
J&J vaccination		−0.005			1.047	
		(0.979)			(0.013)	
J&J vaccination, instrumented			−0.247			3.190
			(0.659)			(0.033)
Other vaccines	−0.299	−0.290	−0.319	0.779	0.781	1.008
	(0.079)	(0.091)	(0.075)	(0.014)	(0.013)	(0.004)

Demographic controls	Yes	Yes	Yes	Yes	Yes	Yes

Observations	251	251	251	127	127	127
*R*-squared	0.118	0.117	0.113	0.195	0.189	0.063
*F*-test value	6.052	5.904		5.922	5.433	

*Note*: Unstandardized regression coefficients are reported. Parentheses contain *p*-values from robust standard errors. The sample size of columns 4–6 is half of columns 1–3 because we split the sample for two randomized experiments: one describing the hypothetical recipient country of US aid as “developing countries” in general, the other as “North Korea.”

Columns 1 to 3 in table 5 show that neither eligibility for J&J vaccination nor instrumented J&J vaccination positively impact views of the United States. None of the coefficients in either specification is statistically significant. Reaffirming previous results, American generosity in donating vaccines did not result in higher favorability toward it.

In contrast, columns 4 to 6 show that the donated vaccines brought about a significant change in the attitude of those who benefited from them: they became more likely to support South Korean vaccine aid to other countries. Although the relatively small sample size of J&J recipients cautions against drawing firm conclusions about the respective coefficients, the results are consistent across a range of specifications. Whether using eligibility (column 4), actual shots (column 5), or instrumented J&J vaccination (column 6) via eligibility, donated shots lead to higher support for vaccine aid.

We also repeat the same analysis for men only. The pool of men eligible for the J&J shot was nearly the entire male population in their thirties and early forties due to their high compliance rate with mandatory military service and automatic transfer after active duty to reserve and civil defense roles. Women, however, had to be employed in defense or foreign affairs fields to be eligible. The disparity in eligibility across genders meant that the vast majority of J&J was given to males, and that the pool of those eligible was much more representative of the population for men.^9^ Thus, we run the same models for only men to rule out any potential bias from including females working in such a specialized field. Columns 7 to 12 all corroborate our results. It should be noted that while the coefficients in these columns also show that J&J’s effect is larger than that of other vaccines, it is misleading to directly compare them, for the coefficient on the “other vaccines” variable is not the estimate from a discontinuity design.^10^

These results of the J&J vaccines contrast with the panel data analysis from previous sections, which are based on all vaccines, and point to a key difference between the effects of commercially procured and donated vaccines. Those on the receiving end of American generosity were more willing to “pay it forward” by backing South Korean vaccine donations to those in need. On the other hand, it is unclear whether those inoculated with American vaccines purchased on the market were more likely to approve of Seoul shipping vaccines overseas. Only with information about US aid to the developing world did they partially get behind Korea sending vaccines abroad. Donating vaccines promotes passing on generosity in ways that selling vaccines does not.

Respondents who had received donated shots were more supportive of sending aid even when the survey question did not specify the number of countries or identify the destination of the aid beyond “developing nations.” Rather than expectations about possible future returns from sending aid, it was likely the donated nature of the shots themselves that led to the willingness to send help to others in need as a form of generalized reciprocity.

## Conclusion

The preceding analysis found no evidence that American vaccines improved views of the United States. Nevertheless, US vaccine aid had two distinct effects relevant to US interests. First, donated vaccines significantly increased support for the Korean government sending vaccines to the developing world. Foreign assistance moved Koreans to “pay it forward” and back their own government in helping others. Second, information about the United States providing jabs to the developing world also increased support for South Korean aid to other countries among vaccinated political moderates. The example that such aid sets spurs public opinion in third-party nations to help others under duress. These two effects, in theory, could be mutually reinforcing.

Robust support for generalized reciprocity in foreign assistance emerged despite the identity and number of potential aid recipients being unknown, making it unlikely that a *quid pro quo* calculation formed the basis for such mass sentiments. Such approval for carrying on goodwill raises prospects of international collaboration through a sequential chain of cooperative behavior across multiple nations.

These findings have policy implications. Vaccines provided via aid, this paper indicates, could mitigate the political pressures that “vaccine nationalism” puts on governments and shore up support for providing vaccines abroad. Afforded the protection that donated jabs provide, citizens become more supportive of sending vaccine aid abroad. Information that the United States is sending aid to the developing world can further increase support for donating vaccines abroad. A more efficient vaccination drive becomes feasible.

The results also point to the need to consider the capacity to deliver future aid when allocating assistance. For example, directing shots to nations with mass vaccine production infrastructure (such as India, Belgium, or South Korea, among others) could alleviate pressure to hoard or block the export of vaccines amidst a shortage, thereby minimizing bottlenecks in the supply chain. Once the vaccine shortage eases, this can also lead to an additional round of vaccine aid being forwarded to third countries, enabling a cooperative approach to future global vaccination drives via a cascade of vaccine aid.

Future research could examine whether the “pay it forward” effect of vaccine aid applies to other forms of aid and how long such support for generalized reciprocity endures. Our analysis shows that two months after receiving donated US vaccines, South Koreans were willing to pass on the generosity by supporting aid to others in need. Testing whether reminders of past help can mobilize present-day support would extend these findings. Regarding the “example effect,” varying information about donor countries offers another promising line of inquiry. Finally, research could assess whether a feedback loop exists, in which awareness that a recipient has passed along goodwill reinforces public support for further giving in the donor country, creating a self-sustaining mechanism of generalized reciprocity.

## Supplementary Material

nfag003_Supplementary_Data

## Data Availability

Replication data and documentation are available at https://doi.org/10.7910/DVN/ZCICMV.

## Footnotes

1 These benefits of widespread vaccination benefit even those who have not received the vaccine. There is a debate about the extent to which vaccination alone can help prevent the mutation of viruses (Van Egeren et al. 2023).

2 Background information on South Korea’s pandemic response and delayed vaccination program is available in Appendix C.

3 US Department of State, COVID-19 Vaccine Donations webpage, https://www.state.gov/countries-areas/south-korea/#covid_map_link, accessed November 19, 2022.

4 For background information on compulsory military service in South Korea, including time of service, exemption rates, and reserve duty, refer to Appendix D.

5 The surveys received approval from the Institutional Review Board at the KDI School of Public Policy and Management (approval number: 2021-08). Outlines of each survey, as well as the wording and order of questions analyzed in subsequent sections, are available in the Supplementary Material. An analysis of the traits of those that were more likely to drop out (women, younger participants, and less educated participants), as well as how neither of our dependent variables (views of US nor support for foreign aid) predicts retention, is included in table B1.

6 Following guidelines from the Korean Disease Control and Prevention Agency (as well as the American CDC), “fully vaccinated” in this paper means having received one shot of the J&J vaccine or two shots of the Moderna, Pfizer, and AstraZeneca vaccines.

7 Korea National Institute of Health, “Update on Corona-19 Vaccines,” September 9, 2022.

8 The survey also asked about favorability for China and Japan. Favorability for other countries could be deteriorating during the pandemic, while for the United States it is remaining stable (partially due to its vaccines). Running the same models but with favorability for Japan and China as the dependent variables, we do not find favorability for either country deteriorating during the two surveys. Additionally, we create a relative favorability measure by subtracting favorability levels of China from those of the United States and then rerun the tests. Again, we find no significant results.

9 As noted in Appendix D, over 95 percent of men serve in some capacity in the South Korean armed forces and then perform reserve and civil defense duties afterward. They were all eligible for the J&J vaccine from the age of thirty until forty. Men are released from civil defense duties once they reach the age of forty-one and hence become ineligible for the vaccine.

10 While we hesitate to make a causal claim about the “other vaccines” variable, for it is not derived from the discontinuity design, we do note that there is additional evidence that vaccination status is correlated with support for Korean vaccine aid, regardless of vaccine origin, in the cross-section specifications in table 3.

## Appendix A. AAPOR-Required Disclosure Elements

**Survey Sponsor and Fieldwork Agency**

The survey was designed by the authors and administered by Embrain, a Korean online research firm.

**Field Dates**

Wave 1: August 6–18, 2021

Wave 2: February 23–March 10, 2022

**Mode of Data Collection**

Online survey using an opt-in internet panel managed by Embrain. Embrain recruits panel members through online advertisements, partner platforms, and web intercepts. Exact compensation level is proprietary. It also manages panel integrity through duplicate checks, inactivity screening, demographic balance monitoring, and exclusion of individuals with a history of low-quality responses.

**Population Represented**

South Korean adults aged 20 to 69. Panel membership requires verification of Korean nationality; thus, the survey represents citizens rather than all residents.

**Sampling Method**

Quota sampling based on gender, age group, and region to approximate the national population.

**Sample Size**

Wave 1: 3,459 respondents

Wave 2: 2,006 of the same respondents completed the follow-up

**Response/Cooperation Rate**

As this was a nonprobability sample using an opt-in panel, traditional AAPOR response rates are not applicable. Respondents were invited based on age, gender, and residential region. The survey closed when quotas for each of the stratified groups were met. A cooperation rate, defined as the proportion of panelists who completed the survey after being invited, was not reported by panel provider. The response rate for the second wave of the panel was 58.8 percent. Some 27 of the respondents from the second wave were deleted by the survey firm after being deemed low-quality responses.

**Weighting**

No post-stratification weights were applied. All analyses are unweighted.

**Handling of Missing Data**

There are no missing data. In the second wave of the survey, 27 respondents were dropped due to low-quality responses.

**Measures of Uncertainty**

Robust standard errors are reported. Due to the nature of the quota sample and lack of design weights, design effects were not estimated.

**Questionnaire Availability**

Full question wording, translations, and response options for the information used in the paper are provided in the Supplementary Material.

**Question Order**

A numbered list of survey items indicating question order is provided in the Supplementary Material.

**Table B1.** Retention pattern.

	(1)	(2)	(3)	(4)	(5)	(6)
Estimation method:	OLS			Logit		
Female	−0.108	−0.112	−0.097	−0.457	−0.475	−0.408
	(0.000)	(0.000)	(0.000)	(0.000)	(0.000)	(0.000)
Age						
20s	−0.175	−0.174	−0.161	−0.732	−0.733	−0.674
	(0.000)	(0.000)	(0.000)	(0.000)	(0.000)	(0.000)
30s	−0.098	−0.099	−0.099	−0.424	−0.429	−0.425
	(0.001)	(0.001)	(0.021)	(0.001)	(0.001)	(0.020)
40s	−0.036	−0.041	−0.055	−0.160	−0.180	−0.236
	(0.028)	(0.028)	(0.040)	(0.124)	(0.126)	(0.174)
50s	−0.025	−0.028	−0.007	−0.109	−0.124	−0.032
	(0.028)	(0.028)	(0.039)	(0.122)	(0.123)	(0.172)
Education						
Some college	0.095	0.095	0.124	0.394	0.396	0.515
	(0.000)	(0.000)	(0.000)	(0.000)	(0.000)	(0.000)
Postgraduate	0.168	0.169	0.171	0.728	0.733	0.723
	(0.000)	(0.000)	(0.000)	(0.000)	(0.000)	(0.000)
Political orientation						
Conservative	−0.007	−0.000	−0.013	−0.031	−0.002	−0.054
	(0.023)	(0.023)	(0.032)	(0.097)	(0.099)	(0.139)
Progressive	−0.006	−0.006	−0.032	−0.023	−0.025	−0.134
	(0.019)	(0.019)	(0.028)	(0.082)	(0.082)	(0.117)
View of US		−0.011			−0.049	
		(0.007)			(0.032)	
Support for			0.003			0.014
vaccine aid			(0.008)			(0.032)
Constant	0.623	0.674	0.582	0.523	0.747	0.351
	(0.000)	(0.000)	(0.000)	(0.000)	(0.000)	(0.131)
Observations	3,459	3,459	1,733	3,459	3,459	1,733
*R*-squared	0.040	0.041	0.040			

*Note*: Unstandardized regression coefficients are reported. Parentheses contain *p*-values from robust standard errors.

**Table B2.** US vaccine aid and attitude toward United States (cross-section analysis).

	(1)	(2)	(3)	(4)	(5)	(6)
Dependent variable:	View of US (1–7)					
Data:	1^st^ wave			2^nd^ wave		
Sample:	All	All	under 60	under 60	All	All
Vaccinated	0.007	−0.045	0.015	−0.089	−0.177	−0.126
	(0.865)	(0.490)	(0.731)	(0.232)	(0.102)	(0.348)
Vaccinated		0.074		0.136		−0.073
v US vaccine		(0.303)		(0.091)		(0.482)
Female	−0.347	−0.345	−0.367	−0.362	−0.368	−0.367
	(0.000)	(0.000)	(0.000)	(0.000)	(0.000)	(0.000)
Age						
20s	−0.110	−0.107	−0.120	−0.113	−0.039	−0.037
	(0.076)	(0.084)	(0.053)	(0.066)	(0.666)	(0.676)
30s	−0.407	−0.401	−0.417	−0.406	−0.361	−0.359
	(0.000)	(0.000)	(0.000)	(0.000)	(0.000)	(0.000)
40s	−0.314	−0.312	−0.320	−0.315	−0.373	−0.375
	(0.000)	(0.000)	(0.000)	(0.000)	(0.000)	(0.000)
50s	−0.020	0.020			0.077	0.040
	(0.791)	(0.816)			(0.432)	(0.717)
Education						
Some college	0.033	0.033	0.085	0.085	−0.063	−0.064
	(0.493)	(0.497)	(0.108)	(0.110)	(0.375)	(0.366)
Postgraduate	0.099	0.098	0.149	0.147	0.055	0.053
	(0.197)	(0.200)	(0.077)	(0.079)	(0.590)	(0.606)
Political orientation						
Conservative	0.598	0.599	0.558	0.558	0.746	0.746
	(0.000)	(0.000)	(0.000)	(0.000)	(0.000)	(0.000)
Progressive	−0.048	−0.047	−0.040	−0.039	0.021	0.022
	(0.284)	(0.295)	(0.389)	(0.402)	(0.727)	(0.724)
Constant	4.530	4.524	4.507	4.499	4.658	4.678
	(0.000)	(0.000)	(0.000)	(0.000)	(0.000)	(0.000)
Observations	3,459	3,459	2,988	2,988	2,006	2,006
*R*-squared	0.093	0.094	0.088	0.089	0.112	0.112
*F*-test value	35.230	32.108	31.338	28.409	24.789	22.581

*Note*: Unstandardized regression coefficients are reported. Parentheses contain *p*-values from robust standard errors.

**Table B2.** (Cont). US vaccine aid and attitude toward United States (cross-section analysis).

	(7)	(8)
Dependent variable:	ΔView of US (1–7)	
Data:	Both waves	
Sample:	All	All
Vaccinated after 1^st^ wave	−0.040	−0.036
	(0.380)	(0.472)
		(0.447)
Age		
20s		0.048
		(0.550)
30s		0.055
		(0.471)
40s		−0.023
		(0.755)
50s		0.015
		(0.869)
Education		
Some college		−0.107
		(0.084)
Postgraduate		−0.045
		(0.616)
Political orientation		
Conservative		0.118
		(0.052)
Progressive		0.052
		(0.339)
Constant	−0.002	0.033
	(0.947)	(0.720)
Observations	1,886	1,886
*R*-squared	0.000	0.005
*F*-test value	0.771	1.049

*Note*: Unstandardized regression coefficients are reported. Parentheses contain *p*-values from robust standard errors.

Appendix B. Additional Analysis and Robustness Checks

Columns 1 and 2 report results of cross-sectional analysis with measures of attitudes toward the United States and vaccine status collected during the first survey. Columns 5 and 6 do the same for the second survey. The cross-sectional study provides informative comparisons between the vaccinated and unvaccinated, since government policy at the time tightly regulated eligibility for the virus. While prioritizing medical staff, employees in essential public services, and first responders, authorities limited access to shots primarily by age, starting with the over-75 group and moving to younger groups in 5- or 10-year increments. By the time of the first survey, nearly 90 percent of those 60 years and older had received their shots, while those under 60 remained between 25 percent and 40 percent, as seen in table 1. Thus, self-selection into vaccination status by unobserved characteristics that might influence their view on foreign affairs for the under-60 group was limited by government policy. Columns 3 and 4 report the results where the sample is limited to those under 60.

The even-numbered columns show the results that differentiate between the effects of vaccines in general (all vaccines including British AZ) and American vaccines (all vaccines excluding AZ). Apart from column 4 (at the *p *= 0.10 level), we find no evidence for American vaccines impacting favorability toward the United States.

Results of the primary analysis using both waves are shown in columns 7 and 8. These specifications compare changes in views of the United States between those vaccinated prior to the first round of the survey and those who received the vaccine between the first and second survey. The estimates for the “vaccinated after first wave” coefficient are not statistically significant, suggesting that vaccination had no causal impact on the views toward the United States.

**Table B3.** Knowledge about the origin of vaccines (percent correct).

“My vaccine came from…”	UK	US	Germany	Don’t know/other wrong answer
Pfizer	6.1	78.6	7.2	11.2
AstraZeneca	66.0	14.7	0.9	18.6
Moderna	4.3	81.1	1.8	12.8
J&J	5.1	79.6	1.3	14.0

**Table B4.** US vaccine aid and attitude toward US: removing the vaccinated with incorrect identification of vaccine’s origin.

	(1)	(2)	(3)	(4)	(5)	(6)	(7)	(8)
Dependent variable:	View of US (1–7)						△ View of US	
Data:	1^st^ wave			2^nd^ wave			Both waves	
Sample:	All	All	Under 60	Under 60	All	All	All	All
Vaccinated	0.058	0.019	0.063	−0.090	−0.137	−0.109		
	(0.209)	(0.814)	(0.192)	(0.362)	(0.217)	(0.449)		
Vaccinated		0.051		0.185		−0.039		
v US vaccine		(0.564)		(0.079)		(0.738)		
Vaccinated after 1^st^ wave							−0.043	−0.021
							(0.388)	(0.705)
Female	−0.354	−0.353	−0.387	−0.384	−0.359	−0.359		−0.021
	(0.000)	(0.000)	(0.000)	(0.000)	(0.000)	(0.000)		(0.678)
Age								
20s	−0.112	−0.111	−0.122	−0.116	−0.032	−0.032		0.071
	(0.087)	(0.090)	(0.062)	(0.074)	(0.727)	(0.733)		(0.395)
30s	−0.405	−0.403	−0.416	−0.407	−0.367	−0.366		0.049
	(0.000)	(0.000)	(0.000)	(0.000)	(0.000)	(0.000)		(0.538)
40s	−0.345	−0.345	−0.352	−0.349	−0.411	−0.411		−0.020
	(0.000)	(0.000)	(0.000)	(0.000)	(0.000)	(0.000)		(0.791)
50s	−0.003	0.023			0.156	0.136		0.087
	(0.967)	(0.815)			(0.148)	(0.267)		(0.393)
Education								
Some college	0.006	0.006	0.051	0.051	−0.091	−0.091		−0.112
	(0.908)	(0.909)	(0.354)	(0.352)	(0.221)	(0.220)		(0.089)
Postgraduate	0.062	0.063	0.117	0.116	0.040	0.039		−0.042
	(0.446)	(0.444)	(0.182)	(0.186)	(0.712)	(0.719)		(0.659)
Political orientation								
Conservative	0.589	0.590	0.559	0.558	0.742	0.743		0.116
	(0.000)	(0.000)	(0.000)	(0.000)	(0.000)	(0.000)		(0.069)
Progressive	−0.042	−0.041	−0.030	−0.028	0.038	0.038		0.060
	(0.376)	(0.387)	(0.542)	(0.568)	(0.557)	(0.556)		(0.292)
Constant	4.561	4.558	4.548	4.541	4.646	4.656	−0.000	0.007
	(0.000)	(0.000)	(0.000)	(0.000)	(0.000)	(0.000)	(1.000)	(0.947)
Observations	3,068	3,068	2,717	2,717	1,789	1,789	1,673	1,673
*R*-squared	0.095	0.095	0.092	0.093	0.118	0.118	0.000	0.006
*F*-test value	32.322	29.394	30.182	27.352	23.329	21.211	0.747	1.091

*Note*: Unstandardized regression coefficients are reported. Parentheses contain *p*-values from robust standard errors.

**Table B5.** Vaccination and opinion on vaccine aid: first-difference.

	(1)	(2)
Dependent variable:	△ Support for vaccine aid	
	All	
Vaccinated after 1^st^ wave	0.143	0.194
	(0.312)	(0.188)
Information cue	0.090	0.063
about US vaccine aid	(0.525)	(0.662)

Demographic controls	No	Yes

Observations	452	452
*R*-squared	0.003	0.027
*F*-test value	0.768	0.945

*Note*: Unstandardized regression coefficients are reported. Parentheses contain *p*-values from robust standard errors.

Appendix C. Background to South Korea’s Pandemic Response

South Korea’s first documented COVID-19 case was in January 2020. Over the next two years, it maintained one of the lowest per capita infection and fatality rates among OECD countries. Scholars attribute this early success to the country’s recent experience with viral outbreaks, robust public–private partnerships, an adaptive health system, strong social norms supporting compliance, and high internet penetration enabling rapid monitoring (Choi 2020; Park, Choi, and Ko 2020; You 2020; Park and Chung 2021). The government capitalized on this record in public messaging, emphasizing low case and death rates compared to peer countries—messaging that likely contributed to the governing party’s 2020 general election victory.^11^

In contrast to its containment record, South Korea lagged in securing vaccines. Success in limiting early spread reduced perceived urgency, delaying procurement compared to other capitals. Prime Minister Jung Se-Kyun later admitted that this early effectiveness contributed to a slow start in securing doses.^12^ Several domestic firms pursued vaccine development, with some reaching trial stages in 2020, but none produced a successful candidate. When trial results for Pfizer, Moderna, and J&J exceeded expectations in late 2020, South Korea had no contracts in place, forcing the government to explain its delay while other countries moved ahead.

Mounting public pressure for mass inoculation led to intensified procurement efforts. Officials were dispatched to vaccine developers’ headquarters, and contracts were eventually secured for multiple vaccines. The first stage of South Korea’s vaccination program launched on February 26, 2021—about three and a half months after rollouts began in the United States and Israel—starting with essential medical personnel, then expanding to long-term care facility workers and residents, first responders, and the immunocompromised.^13^ In May, the general population became eligible by age priority, but as late as July 2021, those under 60 remained ineligible for shots, and many adults were uncertain when they could be vaccinated.

The lag in vaccine availability became the central theme of opposition criticism. Unlike in some countries, partisan divides centered not on skepticism about vaccines but on accusations of government mismanagement. In December 2020, the main opposition party charged the government with “incompetence and wrong response leading to problems with supply and delays.”^14^ By mid-2021, frustration persisted, with former presidential candidate Ahn Cheol Soo giving the government “a failing grade” for its vaccine procurement performance.^15^

Appendix D. Background to Military Service in South Korea

South Korea maintains universal male conscription due to ongoing security concerns on the Korean Peninsula. Most men enter active duty in their early twenties, serving between 18 and 21 months depending on the service branch. Upon completing active duty, men are automatically transferred to the reserves for eight years, during which they participate in annual training sessions. Afterward, they serve in the civil defense corps until the age of 40, with responsibilities for local security and emergency response.

The average annual percentage of Korean men exempt from military service during 2003–2021 is 2.2 percent, with the lowest being 1.6 percent in 2013 and the highest being 2.9 percent in 2018. Most of those among the 2.2 percent exempt from active duty are still required to provide civil support services during a war. The average annual percentage of men who are exempt from even civil service duties during wartime is about 0.3 percent.^16^

At any given time, the reserve force includes roughly 538,000 personnel, while the civil defense corps comprises approximately three million. This system means that the overwhelming majority of men in their thirties and early forties are subject to ongoing defense obligations, making them a clearly defined group for targeted policies.

Eligibility for the June 2021 Johnson & Johnson (J&J) vaccine donation was tied to these defense-related obligations. Men in the reserves or civil defense met the criteria, while women were eligible only if employed in specific defense-related or foreign-affairs positions, representing a small share of recipients. The government also excluded those under 30 from receiving the J&J vaccine due to concerns about rare clotting side effects associated with adenovirus-vector vaccines.

Active-duty personnel were already scheduled to receive other vaccines through separate government channels, meaning the donated J&J doses went primarily to reservists, civil defense members, and a limited number of civilians in eligible professions. These combined rules—defense-sector linkage, age cutoff at 30, and exclusion of active-duty—produced a predictable and sharply defined treatment group for the purposes of this study.
