# Supplementary material for: Paying It Forward: Generalized Reciprocity in Mass Opinion on Foreign Aid
Source: Public Opin Q. 2026 Mar 19;90(2):275–309. doi: 10.1093/poq/nfag003 (PMC13081207; doi:10.1093/poq/nfag003)
Supplement: nfag003_Supplementary_Data [file nfag003_supplementary_data.pdf]

Supplementary Material

Paying It Forward:
Generalized Reciprocity in Mass Opinion on Foreign Aid

Joonbum Bae (j.bae@colostate.edu)

Colorado State University

Changkeun Lee (cklee@kdischool.ac.kr)

KDI School of Public Policy and Management

Contents:

Text S1: Outline of Wave 1 Questionnaire

Text S2: Wording and Order of Wave 1 Questionnaire

Text S3: Outline of Wave 2 Questionnaire

Text S4: Wording and Order of Wave 2 Analytic Items

Text S1: Section-Level Outline of Wave 1 Questionnaire

The Wave 1 questionnaire administered by Embrain consisted of multiple modules covering political attitudes, COVID-19 perceptions, vaccination behavior, policy preferences, risk preferences, mental health, and demographic characteristics. The questionnaire was originally fielded in Korean; the outline below is based on the English translation prepared for documentation and replication. Only the items used in the analyses are reproduced in full in Appendix B. The section-level outline is provided to clarify the broader questionnaire context and potential order effects.

1. Screening Module (SQ1–SQ6-1)

This module included basic screening and eligibility questions used to define the target population. Items covered: gender (SQ1), year of birth (SQ2), region of residence (SQ3), general health and height/weight (SQ4–SQ5), and chronic disease diagnosis and timing (SQ6, SQ6-1).

(Analytic variables used: gender, age)

2. Political Orientation and Foreign Attitudes (Q1-1 – Q1-3)

This section measured baseline political predispositions and foreign-country favorability.

Items included: a 7-point political ideology scale (Q1-1), feeling-thermometer ratings toward five countries including the United States (Q1-2), and use of social media for accessing news (Q1-3).

(Analytic variables used: political orientation, U.S. favorability)

3. COVID-19 Risk Perception and Personal Experience (Q2-1 – Q2-13)

These items assessed respondents' views about COVID-19, illness experience, behavioral changes, and economic stress.

Topics included: perceived severity relative to influenza (Q2-1), COVID-19 knowledge (Q2-2), past infection history (Q2-3), contact frequency and mobility (Q2-4 – Q2-10), preventive behaviors (Q2-11), and perceived changes in productivity and income (Q2-12 – Q2-13).

(Analytic variables used: support for vaccine aid, Q2-13)

4. Trust in Government and Evaluation of COVID Policies (Q3-1 – Q3-4)

Items covered evaluations of national health authority performance, major policy tools, transparency, and information access.

(Not used in the present analyses)

5. Policy Preference and Tradeoff Scenarios (Q4-1 – Q4-6)

This module examined preferred levels of social-distancing strictness and evaluated tradeoffs between mortality and economic activity using scenario-based items.

(Not used in the present analyses)

6. Vaccination Experience, Knowledge, and Risk Perception (Q5-1 – Q5-22)

This section collected detailed information on vaccine status, knowledge, attitudes, and experience. Items included: perceived infection probability (Q5-1), vaccination status and type (Q5-2), knowledge of vaccine origin (Q5-3), vaccination timeline and side effects (Q5-4 – Q5-19), and prioritization/ethical evaluations (Q5-20 – Q5-22).

(Analytic variables used: vaccination status, knowledge of vaccine origin)

7. Risk Preferences (Q6-1 – Q6-6)

Multiple price-list choices involving risk and certainty.

(Not used in the present analyses)

8. Time Preferences (Q6-7 – Q6-12)

Intertemporal tradeoff tasks comparing immediate vs. delayed payments.

(Not used in the present analyses)

9. Prosociality, Reciprocity, and Social Preferences (Q6-13 – Q6-17)

Measures of reciprocity, fairness, willingness to punish unfairness, and altruistic behavior.

(Not used in the present analyses)

10. Mental Health Measures (Q7 – Q8)

Standardized mental-health scales (GAD-7 and PHQ-9).

(Not used in the present analyses)

11. Well-Being, Life Satisfaction, Trust, and Demographics (Q9 – Q11-7; DQ1–DQ11)

This section included measures of subjective well-being, affect, trust, social support, and detailed demographic and socioeconomic indicators such as occupation, education, marital status, income, assets, and housing.

(Analytic variables used: education, DQ3)

Note

This outline summarizes the structure and ordering of the full Wave 1 instrument. The complete English translation and original Korean questionnaire are available from the authors upon request.

Text S2: Wording and Order of Wave 1 Questionnaire

Below is the exact wording of the dependent, independent, and control variables used in the analyses, translated from the original Korean questionnaire. Response options are shown as fielded.

Dependent Variables

(Favorability Toward the U.S.)

Q1-2. How favorable do you feel toward each of the following countries? [1 item per row; 7-point scale]

United States: 1 = Very unfavorable, 7 = Very favorable

(Support for Vaccine Aid)

Q2-13. To what extent do you agree or disagree with the following statement:

"Korea should provide vaccine aid to developing countries for humanitarian purposes."

Scale: 1 = Strongly disagree, 7 = Strongly agree

Key Independent Variables

(Vaccination Status)

Q5-2. Have you been vaccinated against COVID-19 (including vaccines received abroad)?

- Pfizer
- AstraZeneca
- Moderna
- Johnson & Johnson
- Other (specify)
- Not vaccinated

(Knowledge of Vaccine Origin)

Q5-3. To the best of your knowledge, what is the country of origin of the vaccine you received?

[Open-ended response]

(Political Orientation)

Q1-1. Where would you place yourself on the following 7-point scale of political orientation?

1 = Very conservative, 7 = Very progressive

Control Variables

(Age)

SQ2. What is your year of birth? [Used to calculate age group: 20s, 30s, 40s, etc.]

(Gender)

SQ1. What is your gender?

- Male
- Female

(Education)

DQ3. What is the highest level of education you have completed?

- No formal education
- Elementary school graduate
- Middle school graduate
- High school graduate
- Associate degree
- Bachelor’s degree
- Graduate degree or higher

Text S3: Outline of Wave 2 Questionnaire

The Wave 2 questionnaire administered by Embrain followed a structure similar to Wave 1 but focused more extensively on updated COVID-19 experiences, vaccination behaviors, and policy evaluations. Only one experimental information treatment and one outcome item were used in the present analyses. The outline below summarizes the structure and ordering of the full Wave 2 instrument.

1. Screening Module (SQ1–SQ3)
This module confirmed respondent eligibility and continuity with Wave 1.
Items covered: gender (SQ1), year of birth (SQ2), and region of residence (SQ3).
(Not used in the present analyses)

2. Health Status and COVID-19 Experience (Q1–Q4)
This section assessed general health, previous COVID-19 infection, contact history, and perceived infection probability.
Topics included: overall health (Q1), past infection diagnosis (Q2), number of infected acquaintances (Q3), and perceived likelihood of future infection (Q4).
(Not used in the present analyses)

3. Vaccination Status, Booster Uptake, and Risk Perception (Q5–Q16)
This module measured detailed vaccination histories and beliefs.
Items included: vaccination completion (Q5), booster uptake (Q6), vaccine type (Q7), knowledge of vaccine origin (Q8), side-effect experiences and perceptions (Q9–Q14), willingness for future vaccination (Q15), and vaccination decisions for children (Q16).
(Not used in the present analyses)

4. Behavioral and Lifestyle Adjustments (Q17–Q23)
This section captured pandemic-related changes in mobility, social interactions, and work patterns.
Topics included: frequency of gatherings (Q17), mobility reductions (Q18), remote work experience (Q19), lifestyle and mental-health changes (Q20–Q23).
(Not used in the present analyses)

5. Information Frames for Policy Evaluation (Q24–Q25)
Respondents were randomly assigned to one of three informational statements about recent COVID-19 mortality or epidemiological trends.

These frames were followed by items evaluating distancing-policy stringency and thresholds for easing restrictions.
(Not used in the present analyses)

6. Evaluation of COVID-19 Policies (Q26–Q29)
This module examined retrospective and prospective evaluations of government responses.
Items covered: assessment of distancing measures, preferred timing for easing restrictions, and willingness to accept case increases under reopening scenarios.
(Not used in the present analyses)

7. Political Orientation and Foreign Attitudes (Q30–Q31)
This section included a 7-point political ideology scale (Q30) and feeling-thermometer evaluations toward foreign countries (Q31), including the United States.
(Not used in the present analyses)

8. Experimental Information Treatment (Q33: Random Assignment)
Immediately before the dependent variable, respondents were randomly assigned to one of four informational cues concerning Korea's or the United States' vaccine-aid activities.
The version used in the present manuscript stated:
"The United States is providing vaccine aid to developing countries for humanitarian purposes."
This treatment appeared directly before the outcome question.
(Analytic variable used: information treatment)

9. Outcome Measure: Support for Vaccine Aid (Q33C)
Following the treatment, respondents rated their agreement with the statement:
"Korea should provide vaccine aid to developing countries for humanitarian purposes."
(7-point scale: 1 = strongly disagree, 7 = strongly agree)
(Analytic variable used: support for vaccine aid)

10. Additional Foreign Policy and Trust Measures (Q34–Q35)
This section included items on U.S.–China strategic rivalry and institutional trust.
(Not used in the present analyses)

11. Personality, Values, Mental Health, and Social Support (Q36–Q50)

This final block included Big Five personality questions, reciprocity, trust, mental-health scales (GAD-7, PHQ-9), well-being indicators, and social-support measures.
(Not used in the present analyses)

Note
This outline summarizes the structure and ordering of the full Wave 2 instrument. The complete translated questionnaire and the original Korean instrument are available from the authors upon request.

Text S4: Wording and Order of Wave 2 Analytic Items

Below is the exact wording of the dependent and experimental variables used in the Wave 2 analyses, translated from the original Korean questionnaire. Response options are shown as fielded.

1. Experimental Information Treatment (Q33: U.S. Humanitarian Aid Frame)
Respondents were shown **one of the four versions listed below**, selected at random with approximately equal assignment probability.
(Programming instruction: "Display one of A/B/C/D at random, ensuring roughly even distribution across respondents.")
Only **version (A) and (C)** are used in the present analyses.

Version (A): Korea → Developing Countries (No U.S. Cue)
"To what extent do you agree or disagree with the following statement?
'Korea should provide vaccine aid to developing countries for humanitarian purposes.'"

Version (B): Korea → North Korea (No U.S. Cue)
"To what extent do you agree or disagree with the following statement?
'Korea should provide vaccine aid to North Korea for humanitarian purposes.'"

Version (C): U.S. Cue → Korea → Developing Countries
"The United States is providing vaccine aid to developing countries for humanitarian purposes.
To what extent do you agree or disagree with the following statement?
'Korea should provide vaccine aid to developing countries for humanitarian purposes.'"
(This is the version used in the present study.)

Version (D): U.S. Cue → Korea → North Korea
"The United States is providing vaccine aid to developing countries for humanitarian purposes.
To what extent do you agree or disagree with the following statement?
'Korea should provide vaccine aid to North Korea for humanitarian purposes.'"

Response Scale (Identical Across All Versions)
1 = Strongly disagree
2 = Disagree
3 = Somewhat disagree

4 = Neither agree nor disagree

5 = Somewhat agree

6 = Agree

7 = Strongly agree

2. Control Variables Used in the Combined Analysis

No additional Wave 2 control variables were used.

Note

The items above are reproduced verbatim in translation from the original Korean Wave 2 questionnaire. The complete translated instrument and the original Korean version are available from the authors upon request.
